# Climate change winners and losers among North American bumblebees

**DOI:** 10.1098/rsbl.2021.0551

**Published:** 2022-06-22

**Authors:** Hanna M. Jackson, Sarah A. Johnson, Lora A. Morandin, Leif L. Richardson, Laura Melissa Guzman, Leithen K. M’Gonigle

**Affiliations:** ^1^ Department of Biological Sciences, Simon Fraser University, 8888 University Drive, Burnaby, British Columbia, Canada V5A 1S6; ^2^ Pollinator Partnership, 600 Montgomery Street, Suite 440, San Francisco, CA 94111, USA; ^3^ Xerces Society for Invertebrate Conservation, 628 NE Broadway, Ste. 200, Portland, OR 97232, USA; ^4^ Marine and Environmental Biology section at the Department of Biological Sciences, University of Southern California, Allan Hancock Foundation Building, Los Angeles, CA 90089-0371, USA

**Keywords:** bumblebees, climate change, land use, species’ declines, occupancy models

## Abstract

Mounting evidence suggests that climate change, agricultural intensification and disease are impacting bumblebee health and contributing to species’ declines. Identifying how these factors impact insect communities at large spatial and temporal scales is difficult, partly because species may respond in different ways. Further, the necessary data must span large spatial and temporal scales, which usually means they comprise aggregated, presence-only records collected using numerous methods (e.g. diversity surveys, educational collections, citizen-science projects, standardized ecological surveys). Here, we use occupancy models, which explicitly correct for biases in the species observation process, to quantify the effect of changes in temperature, precipitation and floral resources on bumblebee site occupancy over the past 12 decades in North America. We find no evidence of genus-wide declines in site occupancy, but do find that occupancy is strongly related to temperature, and is only weakly related to precipitation or floral resources. We also find that more species are likely to be climate change ‘losers’ than ‘winners’ and that this effect is primarily associated with changing temperature. Importantly, all trends were highly species-specific, highlighting that genus or community-wide measures may not reflect diverse species-specific patterns that are critical in guiding allocation of conservation resources.

## 1. Introduction

Whether insect populations are experiencing global declines is a topic of current debate [1–3]. bumblebees are widespread, charismatic and provide critical pollination services to many different flowering plants [4–6], and thus their population trajectories are among the most well studied of wild insects [7–11]. Species’ trends differ [10], with some in serious decline (e.g. *Bombus occidentalis*, *Bombus affinis* [12,13]) and others increasing (e.g. *Bombus impatiens*, *Bombus cryptarum* [14,15]).

To combat biodiversity loss, it is paramount that we identify the species-specific environmental drivers of range expansions and contractions. Several recent studies have made such progress for bumblebees. Hemberger *et al.* [11] found that increasing cropland extent and decreasing crop richness were associated with bumblebee species’ declines in the Midwest, USA, but trends varied among species. Specifically, occurrences of *B. terricola*, *B. fervidus* and *B. borealis* declined with cropland extent, while occurrences of *B. affinis*, *B. bimaculatus* and *B. impatiens* increased. Similarly, Cameron *et al.* [7] found that pathogen load was correlated with potential declines in *B. occidentalis*, *B. affinis* and *B. pensylvanicus* in North America. Previous studies have also shown that species’ differ in their responses to climate change [16,17], with some showing contracting southern range limits [16].

Making general conclusions at larger spatial scales is complicated by the fact that inferred species’ trends can differ greatly across studies. Consequently, large-scale analyses must quantify how modelling assumptions may bias parameter estimates. For example, Soroye *et al.* [9] used occupancy models to assess species’ declines and potential links to climate change in North America and Europe, however, subsequent analyses identified modelling assumptions that led to greatly overestimated declines in that work [10]. Identifying drivers of species-specific declines or increases remains an important and open question.

Evaluating determinants of suitable habitat, such as landscape features or climatic conditions (e.g. temperature, precipitation) is critical if we hope to accurately predict how species’ responses to environmental change may shift through time and space, and take action to mitigate effects. Under climate change, some species (the ‘winners’) will experience range expansions, while others (the ‘losers’) will experience range contractions [18,19]. Species distribution models have helped identify the environmental covariates that determine the extent of species’ ranges [20] and are often used to predict future ranges under different climate change scenarios (e.g. [17,21,22]). For bumblebees, in particular, Suzuki-Ohno *et al.* [23] used species distribution models to link past changes in ranges to changes in temperature and land use. While these are important advances, species distribution models do not account for detection bias and, thus, are not ideal for analysing large-scale historical datasets where such detection bias has likely changed through time.

Occupancy models estimate a species’ probability of occurrence across a set of sites and have been used to model associations between bumblebee presence and various landscape and climate characteristics [1,24,25]. Such presence/absence data are not as information-rich as abundance data, and it is possible for the two metrics to exhibit conflicting patterns. While species occurrence is often correlated with abundance, the simple binary structure of occupancy data makes it less sensitive to sampling methodology and thus, more appropriate for analyses of datasets that comprise multiple sources. The power of occupancy models is their ability to explicitly account for biases in species’ detection probability that, if ignored, can lead to spurious inferences. This makes them ideal for analysing data that are aggregated from multiple sources and, consequently, where detection biases can vary across sites, through time, or with environmental variables. However, these models are only effective if they include the right predictors, so if we miss important fixed or random effects, our estimates of occupancy may still be biased.

While occupancy models are typically applied to the presence/absence data, recent studies have shown that their application to presence-only data is possible [10,26–29]. In addition, *multi-species* occupancy models that account for species’ expected ranges have been shown to be relatively effective at estimating species-specific trends over large timescales [10,30]. Here, we apply occupancy models to a large bumblebee dataset to identify temporal drivers of change over the last century in North America. We explore the impacts of changing temperature, precipitation and floral resources on bumblebee site occupancy. Specifically, we ask two questions:

Q1 Is there evidence for genus-wide declines in bumblebee site occupancy in North America over the past 120 years and, if so, how much does this trend characterize species-specific responses?
Q2 Are species-specific changes in occupancy linked to changes in temperature, precipitation and/or floral resources?

## 2. Methods

### (a) Data Sources

#### (i) Bumblebee records

We use a large continental-wide bumblebee dataset [31] that, before any filtering, comprises 649 407 specimen records from 46 species and spans 1805–2020. These records have been compiled from a variety of collections and sources with reputable origin. We filter the above entries to only contain those with unique combinations of species, coordinates, date, and observer, and remove records collected prior to 1901, that are outside North America, or that are incomplete (e.g. missing coordinates, year, environmental data). We also remove species that have poor data coverage or are newly described (*B. cockerelli* and *B. kluanensis*). In addition, we also exclude sites (defined below) that do not contain records in at least two eras (defined below) and sites that are not observed in multiple time intervals (also defined below) in at least one of these eras. After all stages of filtering, our final dataset contains 235 621 unique records. We also run our models with stricter data filtering, wherein we required each site to contain five observations in each 20-year era (removing 1541 sites while keeping 254, which removes 91 633 records and keeps 149 955). These stricter filtering steps help identify the extent to which sites or species with few observations might influence our conclusions; it turns out to not qualitatively impact our conclusions (see electronic supplementary material, figure S1). We also run models where we constrain to only records collected on or after 1960 and this also did not change our conclusions (see electronic supplementary material, figure S2 and table S5).

To construct sites, we overlay a grid across North America. We consider three spatial resolutions: 50 × 50 km, 100 × 100 km and 250 × 250 km and we present results for 100 × 100 km in the main text. We split records (which span 1901–2020) into six 20-year ‘eras’, each of which we further divide into four 5-year ‘time intervals’ wherein, at each site, a bumblebee species could have been observed (detection = 1) or not observed (detection = 0). We use 5-year time intervals because 5 years is short enough to ensure that we have a sufficient number of intervals in each era, but large enough that we minimize the number of intervals with no observations. We infer non-detections (detection = 0) by identifying site × time interval combinations where visits to sites to collect or observe bumblebees were known to have occurred. If *any* bumblebee species had been detected at a given site during a given time interval, we assume that other bumblebees, if present, could also have been detected and thereby set detection status to 0 for those species [26]. In addition, we only model each species over the sites that we infer to be plausibly within their respective ranges, which we construct for each species by tracing a convex hull around all sites containing observations of that species, regardless of when they occurred [10].

#### (ii) Site level environmental predictors

We compile climatic variables using CHELSA high-resolution climate data for earth [32,33]. We calculate the mean monthly maximum temperature and the mean precipitation at a given site in a given era. We also quantify floral resources for bees by combining classifications of land use estimates for the Holocene (HYDE) [34] with previously established floral resource scores for bees [35]. We overlay the HYDE land-use map with the CDL (crop data layer) map to obtain the categories of the CDL that geographically overlap with the HYDE categories for 2008. Then for each HYDE category, we calculate the average floral resources reported by Koh *et al.* [35]. Koh *et al.* [35] provide expert-opinion derived floral resource scores for many types of crops and other land-use categories and we average these for the various land-use types within each site in each era. The temporal variation in floral resources in our dataset, therefore, arises from variation in land use through time in HYDE and not variation in floral-resource values for each category. We sum floral resources across spring, summer, and autumn to provide an overall metric through the season. We denote our floral resources by *FR* and, while the actual magnitude of these scores are not particularly meaningful, relative values between sites are. In our dataset, our floral resource scores range from 0.80 *FR* to 1.61 *FR*. We calculate predictor values for each site in each era.

### (b) Occupancy models

We develop the first multi-species occupancy model for bumblebee occurrence records in North America that directly estimates effects of climate and land-use variables on species’ occupancy. In constructing our models, we build on work done by [30] that tested the validity of various methods of applying occupancy models to large-scale presence-only datasets. Here, we present two models: one where time is a predictor of occupancy (Era model) and the other where climate and land use are predictors of occupancy (Environmental model). Full model details and parameter definitions are provided in the electronic supplementary material and we provide a short summary here.

*Era model:* To test for genus-wide temporal trends in bumblebee occupancy (Q1), we consider a simple model wherein we model the effect of ‘era’ as a direct predictor of each species’ occupancy, letting *μ*_*ψ*era_ denote the mean effect across all species and *ψ*_era_[*i*] denote the effect for species *i*.

*Environmental model:* Next, we replace the effect of era in the above model with environmental predictors that vary across sites and eras (Q2). Specifically, we include linear and quadratic effects of site-averaged maximum temperature (mean linear effect across species denoted by *μ*_*ψ*temp_, standard deviation by *σ*_*ψ*temp_, and species-specific responses by *ψ*_temp_[*i*]; quadratic effect denoted by *ψ*_temp2_), a linear effect of site-averaged precipitation (analogously denoted by *μ*_*ψ*precip_ *σ*_*ψ*precip_, *ψ*_precip_[*i*]), and a linear effect of site-averaged floral cover (analogously denoted by *μ*_*ψ*floral_ *σ*_*ψ*floral_, *ψ*_floral_[*i*]). The quadratic effect of temperature allows the model to estimate each species’ thermal optima from which deviation in either direction leads to decreases in occupancy. To minimize model complexity, we only estimate a single community-wide quadratic effect of temperature, rather than species-specific quadratic effects. In doing so, we are assuming that all species have approximately the same niche breadth, while still allowing for species-specific responses to temperature. We do not include era in this model because variation in occupancy due to any monotonic increase or decrease in environmental covariates would then be accounted for by this non-environmental temporal variable. However, when we did include all variables in a single model, our conclusions did not change (electronic supplementary material, figure S3).

In both of the above models, we model detection probability with a site- and era-specific random effect. This allows detection to vary relatively independently across sites and between eras. We also considered models that included an additional fixed effect of era on detection and, again, our conclusions did not change (electronic supplementary material, figure S4).

All code required to replicate this analysis can be found at https://github.com/Hanna-Jackson/bumble-bee-climate.

## 3. Results

Our final dataset contained 235 641 bee records across 1058 sites which translated into 41 174 unique species × site × time interval combinations. Records were biased in both time and space, with the majority of species’ detections in the most recent eras (electronic supplementary material, figure S5). Site–era combinations were well sampled with each receiving, on average, 1.8 visits across the four time intervals (electronic supplementary material, figure S6) and 9.5 positive species’ detections (electronic supplementary material, figure S5) across 3.8 species (electronic supplementary material, figure S7).

**Figure 1.**
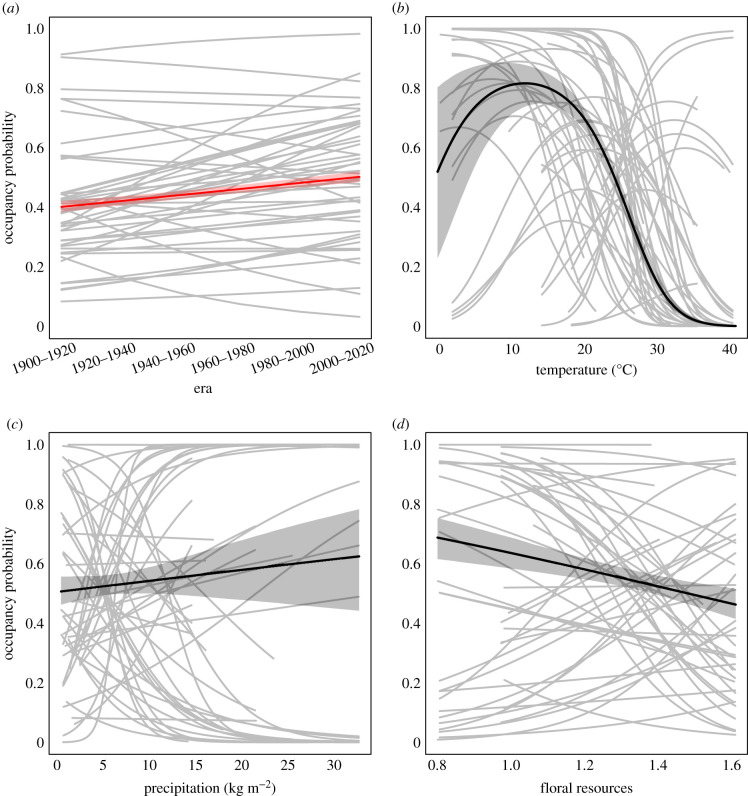
Species-specific occupancy trends are variable but, on average, increase through time (*a*), peak at intermediate temperature (*b*), and are highly variable as a function of precipitation (*c*), and floral resources (*d*). In all cases, species-specific trends (grey curves; only shown over the range of values experienced by that species) are variable and not well characterized by the genus-level trajectories (black lines). Shaded regions denote 95% Bayesian credible intervals. Output in (*a*) is from the Era model and (*b*–*d*) the Environmental model. To highlight that these are two separate models we have plotted the mean line(s) for the Era model in red and the Environmental model in black.

Across bumblebee species, mean occupancy has slightly increased over the past 12 decades (*μ*_*ψ*era_ = 0.081, 95% BCI = [0.019, 0.145], figure 1*a*). However, species-specific trajectories varied, with many showing comparatively large increases or decreases (table 1). Of the 46 species studied, six decreased through time and 22 increased, with the remaining 18 stable (95% BCI around *ψ*_era_[*i*] includes zero; table 1). In our model run where we constrain analysis to observations from 1960 on, our results do not greatly differ (electronic supplementary material, figure S2). For a few species, trends shift from positive or negative to essentially zero, but no species convincingly change directions altogether (electronic supplementary material, table S5).

**Table 1.** Model estimates of species-specific coefficients for *ψ*_era_ (Era model) and *ψ*_temp_, *ψ*_prec_, *ψ*_floral_ (Environmental model). Values in parentheses show 95% Bayesian credible intervals for each estimate and are only included when intervals do not include zero. Positive values indicate higher occupancy at larger values of the corresponding predictor. ‘W’ or ‘L’ indicates a ‘winner’ or ‘loser,’ as defined below in figure 2. In addition, we included IUCN average change, estimated proportional change in occupancy between the first and last era, and *ψ*_era_. IUCN does not report change for species that are not declining, however, we used their method to calculate increases for non-declining species.

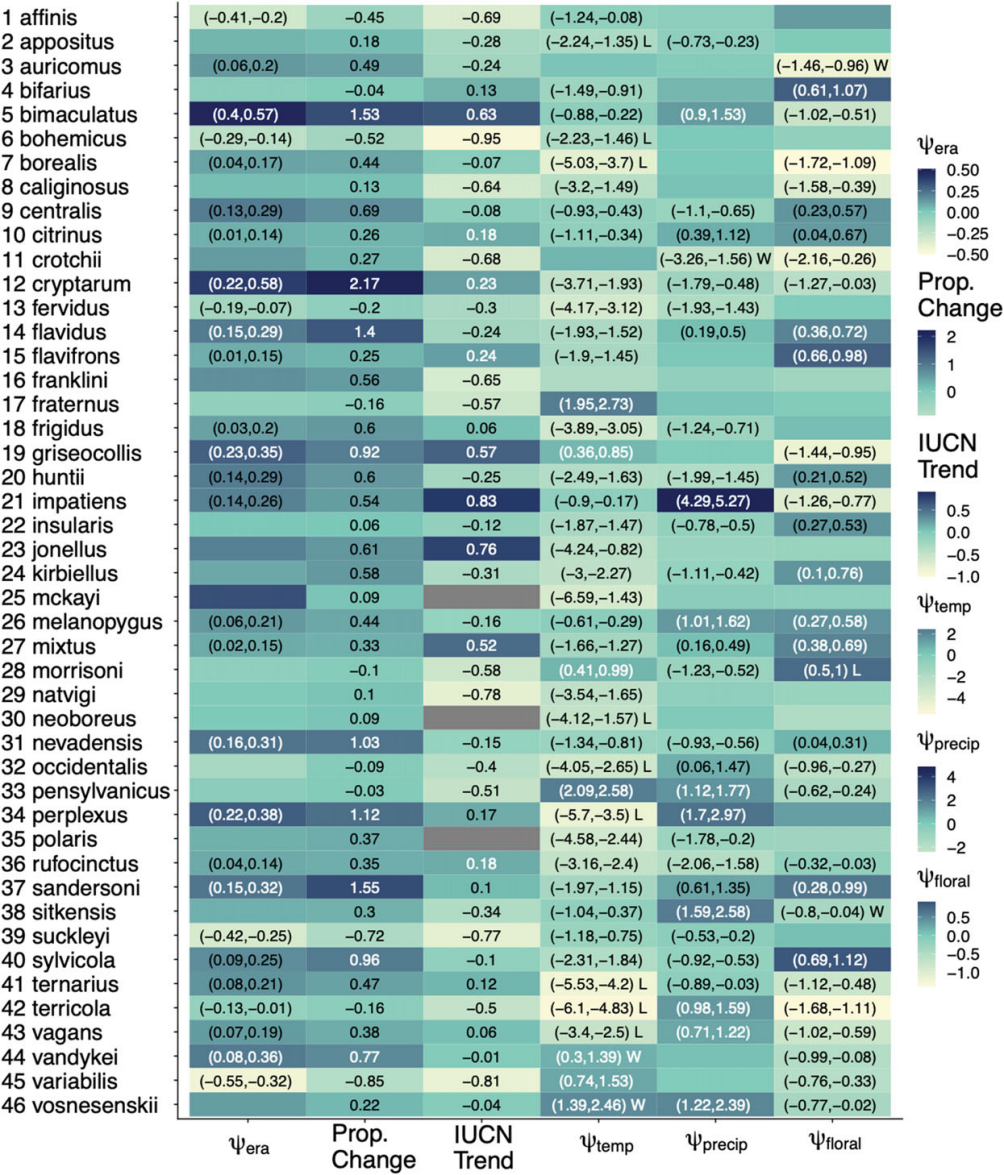

Community-wide occupancy peaks around a mean monthly maximum temperature of $12^{\circ }C$, and was lowest overall at warm temperatures (*μ*_*ψ*temp_ = −1.574, 95% BCI=[−2.168,−0.965], *ψ*_temp2_ = −0.303, 95% BCI=[−0.351,−0.250] figure 1*b*). Again, however, this pattern did not characterize all species, with some exhibiting a higher probability of occupancy at warmer temperatures (table 1). Effects of precipitation (*μ*_*ψ*precip_ = 0.046, 95% BCI=[−0.356,0.446], figure 1*c*) and floral resources (*μ*_*ψ*floral_ = −0.171, 95% BCI=[−0.396,0.044], figure 1*d*) were weaker and, again, species exhibited both positive and negative responses to each (table 1; electronic supplementary material, table S2). Complete model parameter estimates are shown in table 1. When we filtered data to only consider sites that had at least five observations in each of the six 20-year eras, these qualitative patterns did not change (electronic supplementary material, figure S1).

**Figure 2.**
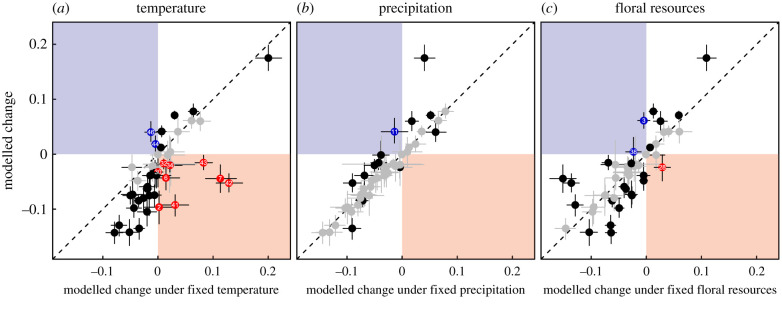
Species’ modelled changes in occupancy under a constant temperature regime (*a*) are mostly more negative than modelled changes when climate variables assume actual values (most points lie below the 1∶1 line), whereas no such pattern exists when changes are modelled under a constant precipitation regime (*b*) or no change in floral resources (*c*). Vertical and horizontal bars denote 95% Bayesian credible intervals and are shaded grey if they overlap the 1∶1 line. Points are coloured black, blue or red if their vertical BCI does not include the 1∶1 line (i.e. if their modelled change in occupancy when all climate variables take actual values is notably larger or smaller than would be expected under a fixed temperature, precipitation, or floral regime). Red colouring indicates species whose century long change in occupancy under constant temperature, precipitation or floral resources are of an entirely different sign than their changes under actual temperature or floral resource change; actual changes have effectively switched these species from ‘winners’ to ‘losers’. By contrast, blue colouring denotes species that have switched from ‘losers’ to ‘winners’ based on observed temperature, precipitation or floral resource change within their ranges. Numbers on blue/red points indicate species identity, as labelled in table 1. Change in occupancy on both axes is computed by calculating the difference between a species’ occupancy in the final era and its occupancy in the first era, with the relevant climate/floral variable held at its mean for calculated values on the horizontal axis. All model coefficients were calculated using our Environmental model, which means changes in occupancy here are entirely a function of temporal changes in environmental variables.

Temperature and precipitation both increased, on average, between 1901 and 2020 (average max temperature by $0.83^{\circ }C$, average precipitation by $0.36\;{\mathrm{kg}\;m}^{-2}\;\mathrm{per}\;\mathrm{month}$), whereas floral resources slightly decreased (by −0.04*FR*) (electronic supplementary material, figure S8). To identify how these changes correlate with species’ trajectories, we compared each species’ estimated environmentally induced change in occupancy between the first and last era to its estimated environmentally induced change over the same period under scenarios where we consecutively held each environmental variable constant at its mean value. Temperature changes had primarily negative impacts, with 37 of the 46 species exhibiting greater declines or less positive increases in occupancy under observed temperature changes than they would have had temperature remained constant (figure 2*a*). By contrast, approximately half of the species were negatively impacted by changes in precipitation (25 of 46 species) or floral resources (24 of 46 species) while the other half were positively impacted (figure 2*b*,*c*). Importantly, nine species exhibit declines that link to changing temperatures within their ranges (red points in figure 2*a*), whereas no such patterns exists for precipitation and only a single species shows such a pattern for floral resources (figure 2*c*,*d*). This difference indicates that changing temperature is likely a major environmental factor driving changes in bumblebee community composition. These findings are largely unchanged for other spatial resolutions and also when using a model that included both era and our environmental predictors, together.

## 4. Discussion

We found evidence that bumblebee occupancy has slightly increased over the past century; however, this genus-level trend poorly summarizes the variable species-specific trends. Our estimated changes in occupancy align closely with IUCN estimates (table 1). For example, both report relative increases in *B. bimaculatus*, *B. griseocollis*, *B. impatiens* and *B. jonellus* and decreases in *B. affinis*, *B. bohemicus*, *B. variabilis* and *B. suckleyi*. However, there are discrepancies (e.g. *B. franklini*, which has not been observed since 2006 [36] and, yet, we estimate to be increasing). Our findings are also consistent with projections from Marshall *et al.* [17] who predicted that some European bumblebee species’ ranges would expand while others would contract with the advancement of climate and land-use change. By contrast, work done in North America and Europe showed that species are failing to track warming at northern range limits and experiencing range losses at southern range limits [16]. Studies looking at North American bumblebee relative abundances have largely found declines, and are generally consistent with our estimates of declines [7,8,11,13].

Our analysis here has a number of important caveats that could lead to the above-noted discrepancies, as well as others. First, our coarse temporal scale of analysis or our decision to model (logit-transformed) linear changes through time are not ideal for species that have undergone recent increases or decreases (e.g. abrupt declines in the 1990s are thought to characterize *B. occidentalis*, *B. affinis*, *B. terricola* and *B. franklini*, [37]). Second, we have attempted to account for temporal variation in detection probability; however, there is likely remaining unmodelled temporal heterogeneity that may bias our estimates of occupancy and thus, estimated trends through time. Third, our Era model and our Environmental model ask related but different questions and reconciling these two models is challenging. Our Era model identifies large-scale temporal trends without providing insight into drivers of change. By contrast, our Environmental model only allows for temporal changes in occupancy to emerge as a consequence of corresponding temporal changes in our environmental variables and actually predicts a mean decline in occupancy over the past 120 years (i.e. the vertical positions of most points in figure 2 are negative). This contradiction indicates that there are likely important temporally variable predictors on species’ occupancy missing from our Environmental model. Identifying such variables is an important direction for future work on this group if we hope to identify drivers of bumblebee change. Given these above caveats, it is important that the documented increase in mean occupancy in our Era model not be extrapolated to imply that bumblebees are on the rise. For example, bumblebee abundance may have decreased over the studied time period; range shifts driven by changing environmental conditions could manifest as increases in occupancy, but concurrent stasis or even declines in abundance [38].

Genus-wide, temperature had notably larger effects than either precipitation or floral resources on bumblebee trends. This result is consistent with Kammerer *et al.* [38], who found that climate variables were more important than landscape factors for predicting abundance and richness of wild bees. It is also consistent with Marshall *et al.* [17], who found that in Europe, both climate and land use were important for determining species ranges, but of the species that are projected to experience range contractions, more loss is projected by climate models than by land-use models. Bumblebees are known to have a variety of adaptations allowing them to be active in cooler conditions [39], and recent work on *B. vosnesenskii* gene expression suggests that cold tolerance may be locally adapted over a species’ range, while the critical thermal maximum is likely invariant [40]. We might expect future warming to further lower site occupancy and, in the long run, drastically modify community composition. Previous work has projected widespread bumblebee range losses under 2050 and 2070 climate scenarios [41] and current range shifts/expansions will have their limits, with some authors expressing concern over whether bumblebees will be able to keep pace with warming [16,42,43].

On average, our model estimated a puzzling slightly negative effect of floral resources on bumblebee occupancy. This may be a consequence of the derivation of our estimates of floral resources; our metric is an aggregate expert opinion rank that attempts to quantify land-use suitability for native bees, in general. To the extent that bumblebee habitat preferences differ from those of native bees, our metric will not reflect bumblebees’ floral resource preferences. Regardless, scoring floral resources at such a large spatial (100 × 100 km) and temporal resolution (20 years), as we have done here, likely does not accurately capture the local resource availability that directly affects individual bumblebees. In addition, our floral metric is less variable than our other environmental metrics, which may limit its ability to explain changes in occupancy (electronic supplementary material, figure S8). Critically, our metric of floral resources also presumes that land-use categories have remained comparable across space and through time over the last century which is likely not always the case. For example, increasing use of herbicides has reduced and delayed flower production in wild plants around monocultures [44] and, in North America, this widespread herbicide adoption occurred between the 1950s and 1970s [45]. Effects of land use on bumblebee occurrence have been difficult to quantify, particularly at this large scale, and here we show that floral resources might not be the ideal metric. For example, Hemberger *et al.* [11] found that crop diversity, rather than crop extent, was a better predictor of changes in bumblebee occurrence and McArt *et al.* [46] found that fungicide usage was a better predictor of bumblebee decline than land use. Lastly, our floral abundance metric was developed with all bee guilds in mind [35] and, thus, may not be appropriate for bumblebees.

Species differed in their responses to all environmental variables and there are some notable trends. For example, *B. impatiens* occupancy increases with higher precipitation which matches previous modelling on that species [14]. *Bombus rufocinctus* and *B. fervidus* exhibited among the strongest negative responses to increased precipitation which also aligns with previous findings that those species associate with dry grassland habitats [47]. Sites or species that experience the most change in temperature are expected to experience the largest changes in occupancy, but looking for evidence of such patterns in raw bee occurrence records is likely to be misleading due to temporal biases in detection probability. Thus, methods that can account for these biases, such as the occupancy models used here, are essential if we hope to generate accurate predictions.

Because environmental variables have changed over the past 120 years, species-specific responses could have led to changes in occupancy. We found that most species’ trajectories, regardless of whether they have increased or decreased over the past 120 years, were negatively impacted by changes in temperature (figure 2*a*). This finding is consistent with similar work on bumblebees from Japan, where temperature emerged as the major factor driving species’ range shifts [23]. Importantly, our model predicts that nine North American species (*B. appositis*, *B. bohemicus*, *B. borealis*, *B. neoboreus*, *B. occidentalis*, *B. perplexus*, *B. ternarius*, *B. terricola* and *B. vagans*), would have benefited from temperature stasis over the last century but, instead, have been negatively impacted by realized changes in temperature. Most of these species have previously been documented as declining, either regionally or on a larger scale [7,8,12,47–49] and, interestingly, most are boreal species (northern, cool-adapted and often forest-associated). This is in line with existing ideas that species with cool-adapted life histories are more susceptible to increasing temperatures [50,51]. Interestingly, six of the nine (*B. bohemicus*, *B. borealis*, *B. perplexus*, *B. ternarius*, *B. terricola* and *B. vagans*) share similar distributions and, thus likely similar climatic preferences, with most observations occurring in the eastern central portion of the continent and extending northwest. *Bombus occidentalis* declines since the 1990s have been well characterized and partly attributed to pathogen spillover from commercially bred colonies [52]. Some unique characteristics of *B. occidentalis* may make it vulnerable to potential synergistic effects of these threats.

Critically, the converse effect of temperature is not true, with only two species, (*B. vosnesenskii* and *B. vandykei*), shifting from expected negative consequences of temperature stasis to estimated benefits of actual temperature change (blue points in figure 2*a*). *Bombus vosnesenskii*’s northward range expansion has been documented, with speculated causes being escape from managed colonies or land-use change [53]. Here, we add an additional hypotheses; namely, that increasing temperature may be a primary driver in this species’ expansion.

Changes in precipitation or floral resources had comparatively minimal effects, with the former not moving a single species from ‘increasing’ to ‘decreasing’ (i.e. no red point in figure 2*b*) and the latter only moving one (*B. morrisoni*). Only a single species, *B. citrinus*, appears to have benefited from changes in precipitation (blue point in figure 2*b*) and only two species (*B. auricomus* and *B. sitkensis*) due to changes in floral resource abundance (blue points in figure 2*c*).

If sampling effort increases through time, simple inferences that are based on raw collection data will likely show erroneous increases in occupancy. Occupancy models like ours use repeated observations to estimate detection probability (roughly, sampling effort) and correct estimates of occupancy for this bias. Compared to analyses of abundance data, our use of binary occupancy data may help reduce bias in our inferences that could stem from increases in sampling effort in recent years. We took additional steps to minimize potential bias in estimates. For example, we constrained inferences for each species to only sites that we inferred to be potentially within that species’ range and, further, only to sites and time intervals where visits to sites were known to have occurred. We let detection probability vary from site to site and between eras, allowing us to account for the fact that some sites were sampled more (or less) thoroughly in recent times. In other words, detection does not have to increase linearly across space or time in our model. In addition, variation in detection probability not accounted for by model predictors may bias our occupancy estimates. For example, changing collector behaviour through time (e.g. if collectors have become more focused on capturing rare species in recent years) or temporal biases in which specimens are inventoried could both impact estimates of occupancy trends. While we believe that our estimates are the best available, given the data we have for bumblebees in North America, species’ estimates of change should not be taken to be definitive. Species-specific, regional studies that assess trends at a more granular spatial and temporal level, such as that by [12], should be prioritized when making management decisions.

Among insect pollinators, bumblebees are one of the most important and effective groups [5,54,55]. Consequently, extinction/colonization dynamics across sites can be expected to have major cascading effects in other flower-associated communities. A large-scale manipulative field experiment, where the most common bumblebee species in each community was removed, found reduced floral fidelity among remaining competitors, demonstrating a potential for cascading, community-dependent impacts of bumblebee species extinctions on plant communities and their visitors [56]. Climate change has been predicted to cause homogenization of functional traits in bumblebees (e.g. proboscis length), which could lead to further major shifts in plant pollination success by interrupting plant–pollinator interactions [57,58]. While climate change may harm some species, others are poised to benefit [59]. Our results show that, for bumblebees in North America, there are very few species that are likely to be such ‘climate winners’. This is highly concerning in a warming world.

## Data Availability

The data used were accessed from: Richardson LL. 2020. Bumble Bees of North America occurrence records database (https://www.leifrichardson.org/bbna.html; accessed 10-07-2020). Data are available from the Dryad Digital Repository: https://doi.org/10.5061/dryad.c59zw3r8f [31]. All code to replicate this analysis can be found at https://github.com/Hanna-Jackson/bumble-bee-climate. Electronic supplementary material is available online [60].
